# ANALYSIS OF INTRINSIC FOOT MUSCLE STRENGTH IN DIABETIC PATIENTS WITH AND WITHOUT DIABETIC PERIPHERAL NEUROPATHY COMPARED WITH HEALTHY CONTROLS

**DOI:** 10.1590/1413-785220263401e289938

**Published:** 2026-02-13

**Authors:** Danielle Moreira de Oliveira, Alan Almeida da Silva, Tarcísio Marconi Novaes Torres, Fabrício Luz Cardoso, Lucas de Menezes Figueiredo, Thiago Batista Faleiro

**Affiliations:** 1 Hospital Universitario Professor Edgard Santos (Hupes-UFBA), BA, Brazil.; 2 Rede Dor - Hospital Sao Rafael, BA, Brazil.; 3 Irmandade Santa Casa de Misericordia de Sao Paulo (ISCMSP), Pavilhao Fernandinho Simonsen, Departamento de Ortopedia e Traumatologia, Sao Paulo, SP, Brazil.; 4 Universidade Federal da Bahia (UFBA), Faculdade de Medicina da Bahia, BA, Brazil.; 5 Hospital Ortopedico do Estado da Bahia (HOE), BA, Brazil.; 6 Hospital Santo Antonio (HSA), Obras Sociais Irma Dulce, BA, Brazil.

**Keywords:** Diabetes Mellitus, Manual Dynamometry, Muscle Strength, Diabetic Neuropathies, Diabetic Foot, Disease Prevention, Diabetes Mellitus, Dinamometria Manual, Força Muscular, Neuropatias Diabéticas, Pé Diabético, Profilaxia

## Abstract

**Objective:**

to evaluate the strength of the intrinsic foot muscles in diabetic patients with and without diabetic peripheral neuropathy, compared to healthy controls.

**Methods:**

a prospective experimental study was conducted with 63 patients divided into three groups: diabetics with peripheral neuropathy (20), diabetics without neuropathy (23), and healthy controls (20). Muscle strength was measured using a digital handheld dynamometer, assessing plantar flexion of the hallux and toes in two positions (neutral and maximum plantar flexion). Statistical analyses were performed using R software, with α=0.05 and β=0.2.

**Results:**

the groups had a mean age of 66 years. Variables with statistically significant differences (p < 0.05) included Right Hallux – Neutral Position, All Toes (Right) – Neutral Position, All Toes (Right) – Plantar Flexion, and Left Toes – Plantar Flexion. Diabetic patients with neuropathy showed lower medians and interquartile ranges in these variables, indicating reduced muscle strength compared to controls.

**Conclusions:**

this study explored the strength of the intrinsic foot muscles in diabetic patients, using dynamometry as an assessment tool. Although no conclusive evidence was found, it is suggested that dynamometry may be useful in the early detection of muscle weakness in diabetic neuropathy. Expanding the study with a larger sample and more data is necessary to validate and refine the preliminary conclusions, contributing to the monitoring of muscle deterioration and the effectiveness of treatments. ***Level of Evidence II; Prospective^d^ comparative study^e^*.**

## RESUMO

**Objetivo:**

avaliar a força da musculatura intrínseca dos pés em pacientes diabéticos com e sem neuropatia periférica diabética, comparados a controles saudáveis.

**Métodos:**

estudo experimental prospectivo realizado com 63 pacientes divididos em três grupos: diabéticos com neuropatia periférica (20), diabéticos sem neuropatia (23), e controles saudáveis (20). A força muscular foi medida utilizando dinamômetro manual digital, avaliando a flexão plantar do hálux e dos artelhos em duas posições (neutra e flexão plantar máxima). Análises estatísticas foram realizadas com software R, utilizando α=0,05 e β=0,2.

**Resultados:**

os grupos apresentaram média de idade de 66 anos. Variáveis com diferenças estatisticamente significativas (p<0,05) incluíram ‘Hálux D - neutro’, ‘Todos D - neutro’, ‘Todos D - flexão plantar’, e ‘Artelhos E - flexão plantar’. Pacientes com neuropatia diabética apresentaram medianas e intervalos interquartis inferiores nessas variáveis, indicando uma redução na força muscular comparada aos controles.

**Conclusões:**

este estudo explorou-se a força da musculatura intrínseca do pé em pacientes diabéticos, utilizando-se a dinamometria como ferramenta. Apesar de não terem sido encontradas evidências conclusivas, sugere-se que a dinamometria pode ser útil na detecção precoce de fraquezas musculares em neuropatia diabética. A ampliação do estudo, com uma amostra maior e mais dados, é necessária para validar e refinar as conclusões preliminares, contribuindo para o monitoramento da deterioração muscular e a eficácia de tratamentos. ***Nível de Evidência II; Estudo^e^ prospectivo comparativo^d^***.

## INTRODUCTION

An epidemic of diabetes *mellitus* (DM) is ongoing. In 1985, it was estimated that there were 30 million adults with DM in the world, this number jumped to 135 million in 1995, reaching 173 million in 2002.^1^ In 2019, the International Diabetes Federation (IDF)^2^ estimated that 9.3% of the global population, aged 20 to 79, had been diagnosed with the disease, this percentage will reach 10.4% by 2040, i.e. 642 million diabetic patients.^3^

About two-thirds of these individuals with DM live in developing countries where the epidemic is more intense, with an increasing proportion of people affected in younger age groups coexisting with the problem that infectious diseases still represent.^3^

The latest data from IDF DIABETES ATLAS^2^ show that Brazil ranks 5th among the countries with the highest prevalence of diabetes: 16.8 million people with diabetes. It is estimated that the prevalence of the disease in the country is 7.6%, with half of these people not aware of having the problem.^4^ It is estimated that this prevalence will increase by almost 50% over the next 25 years.^5^ These figures are worrying, especially because the disease raises mortality by more than 50% compared to the general population of Brazil.

According to the World Health Organization, diabetic foot is characterized by infection, ulceration and/or destruction of deep tissues, associated with neurological abnormalities and varying degrees of peripheral vascular disease in the lower limbs.^6^ Peripheral neuropathy, defined as the presence of symptoms and/or signs of peripheral nerve dysfunction in people with diabetes after excluding other causes, affects almost 50% of patients after 10 years of disease, being the most common complication of diabetes *mellitus*.^3^ Peripheral arterial disease, along with neuropathy, are responsible for the "Diabetic Syndrome", which includes ulcer, Charcot arthropathy, infection and can lead to amputation^7^.

Peripheral vascular disease is the main factor related to the evolution of a diabetic foot ulcer, and it should be diagnosed through a clinical examination of the feet, evaluating the color, skin temperature, pulse palpation and measurement of ankle pressure.^7^

About 40% to 60% of non-traumatic amputations of lower limbs occur in diabetic patients, with 85% of these preceded by foot ulcers.^7^ Skin and soft tissue infections represent a significant impact for both diabetic patients and healthcare systems. Diabetic patients often face longer hospitalization times, high rates of clinical failure, increased readmission rates, and higher mortality rates compared to nondiabetic patients.^8^

When analyzing the investments in the area, it is estimated that around US$760 billion are spent globally annually on DM and its complications, and there are projections that by 2030 the value will exceed US$825 billion.^9^

This study aims to investigate possible changes in the strength of the intrinsic musculature of the foot in diabetic patients using dynamometry. The aim is to test the hypothesis that the loss of strength can serve as an early marker for complications associated with diabetes, such as deformities, ulcers and amputations. This approach aims to offer a strategy to monitor the deterioration of muscle strength and evaluate the effectiveness of the treatments applied. The relationship between muscle weakness monitoring and treatment effectiveness is based on the assumption that early detection of muscle weakness can reflect the progression of diabetic complications and thus allow for more effective adjustments in treatment to prevent adverse outcomes.

## MATERIALS AND METHODS

This project is part of the research "Analysis of the clinical-epidemiological profile of patients undergoing outpatient follow-up in the Department of Orthopedic and Traumatology of University Hospital Prof. Edgard Santos-UFBA", approved with CAAE number 13790619.6.0000.0049.

This prospective experimental study was conducted at the Ambulatory Hospital Complex Professor Edgard Santos, with the aim of comparing diabetic patients, with and without diabetic peripheral neuropathy, with non-diabetic patients. The study exclusively included adult women, aged between 60 and 70 years, recruited from the services of Endocrinology and Orthopedics.

- Inclusion Criteria: The choice to include women between the ages of 60 and 70 was motivated by two main factors:
  1. Homogeneity of the age group: the age range from 60 to 70 years was selected to ensure the homogeneity of the group in terms of age, minimizing the impact of aging-related variables on the results.
  2. Female focus: only women were chosen to be included to reduce the variability associated with gender differences in the prevalence and clinical presentation of diabetic peripheral neuropathy. Studies indicate that women in this age group are more susceptible to diabetes-related complications, justifying the focus of this study.
- Non-Definition of Type of Diabetes (DM I or II): The type of diabetes (I or II) was not specified among the participants, as the central objective of the study is to investigate the presence and absence of diabetic peripheral neuropathy as the main variable. Regardless of the type of diabetes, peripheral neuropathy can develop, and the study's interest lies in comparing outcomes related to this condition.
- Duration of Disease Progression and Degree of Control: Although the duration of diabetes progression and the degree of glycemic control are important factors in the progression of diabetic complications, including peripheral neuropathy, these aspects were not directly controlled in this study. The inclusion of patients who have reached the age of 60-70 years without taking into account these variables is intended to reflect a broader and generalizable clinical scenario.

After clinical evaluation and application of inclusion and exclusion criteria (exclusion of patients with foot deformities, Charcot foot, prior history of amputation or diabetic foot ulcer), 63 patients were selected and divided into three groups:

1. 20 diabetic patients with diabetic peripheral neuropathy;
2. 23 diabetic patients without diabetic peripheral neuropathy;
3. 20 non-diabetic patients, no foot and ankle pathologies.

The selection of the 63 individuals was made from a larger group of patients followed in the services of Endocrinology and Orthopedics, ensuring the representativity of the groups and the validity of the results.

In this sense, the clinical evaluation in the outpatient clinic, in which the Term of Free and Informed Consent (TCLE) was presented for eligible patients. Therefore, after reading the TCLE for the participants and making the clarifications, the signing of the same, as well as the completion of a questionnaire with basic cadastral data was carried out.

The physical examination included a clinical evaluation and tactile sensitivity tests in diabetic patients using the Semmes-Weinstein 10g monofilament.^10^ Six points were evaluated in the plantar region of the right and left feet: hallux, 3rd and 5th toes (plantar region of the distal phalanges) and 1st, 3rd and 5th heads of the metatarsus. The patient's inability to feel the 10g filament at one or more of these points indicated loss of protective sensitivity (LPS), which classified the patient in the group of diabetics with peripheral neuropathy. Diabetic patients who did not experience loss of sensitivity were included in the group of diabetics without peripheral neuropathy. To ensure the accuracy of the results and the reliability of the comparison, similar tests were performed in the group without diabetes and without foot and ankle pathologies.

**Figure 1:**
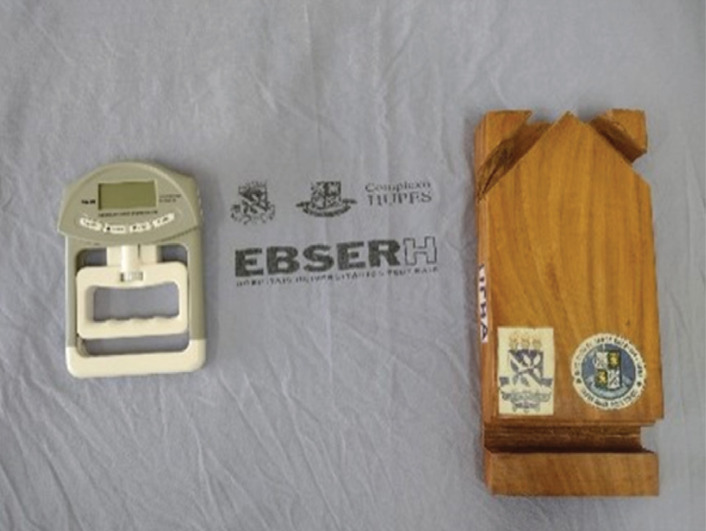
Digital manual dynamometer and an adapted wooden platform.

Source: Author.

- Control and description of deformities: During the physical examination, a check was performed to identify and describe any deformities in the feet, both in diabetic patients and in healthy adults. Deformities were recorded, such as clutched feet or other anatomical alterations, which could interfere with monofilament sensitivity. This documentation helped ensure that the detected loss of sensitivity was not attributable to pre-existing structural deformities.
- Muscle Strength and Dynamometer Test: The contraction force of the intrinsic musculature of the feet was measured using a digital manual dynamometer Bticx^®^ and an adapted wooden platform (Figure 1). The wooden platform provided a stable base during the measurement and ensured the consistency of the tests. The footer on the platform, which mimics the movement axes of the lateral fingers and the hallux, has been designed to facilitate the correct positioning of the feet and ensure that the force applied is measured accurately and repeatably. The dynamometer was positioned to evaluate the plantar flexion of the metatarsophalangeal joints of the hallux and the 2nd, 3rd, 4th and 5th arthritis, as well as the total strength of all arthritis of the right and left feet.

The method of measuring intrinsic muscular strength of the foot using manual dynamometer was described and validated by Xu et al.^11^ and Błażkiewicz et al.^12^ Additionally, Ribot-Ciscar et al.^13^ discuss the application of similar techniques to diabetic patients. To ensure that only the strength of the intrinsic foot muscle is measured, the test was designed to minimize the influence of the leg muscle, with the platform and the dynamometer adjusted to isolate the strength of the foot muscles.

The dynamometer used in our study is the Bticx^®^ handheld digital dynamometer, is certified for use in strength tests, has precise caliber cable and is used to measure grip strength in a variety of applications.

**Figure 2:**
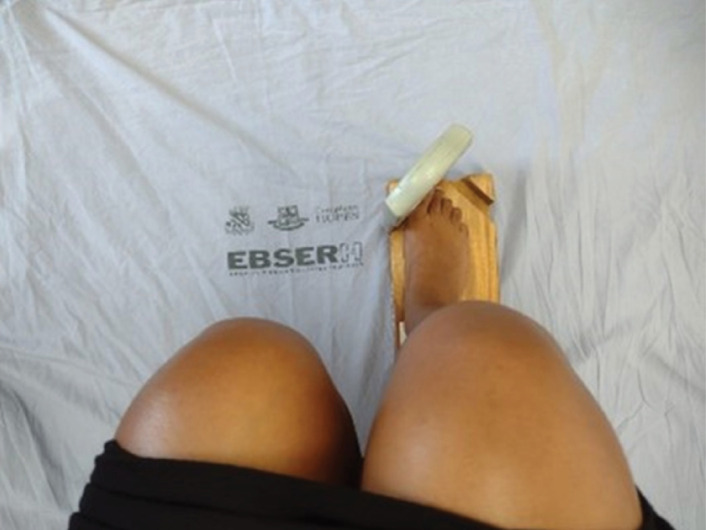
Strength measurement, with ankles in neutral position.

Source: Author.

**Figure 3:**
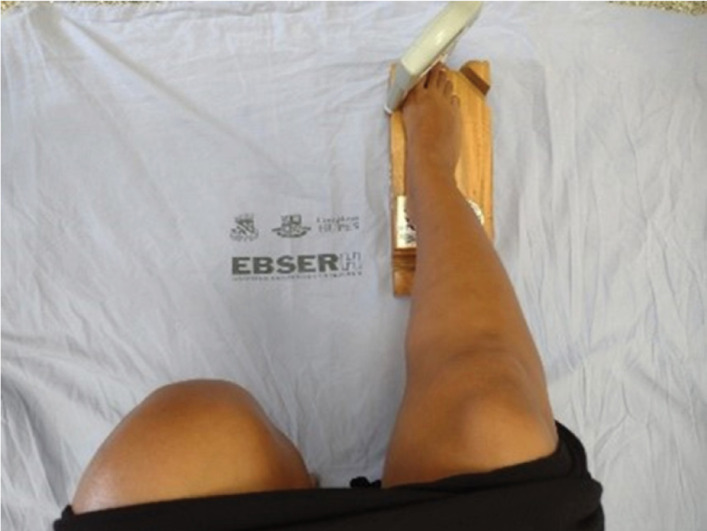
Strength measurement, with ankles in maximum plantar flexion position.

Source: Author.

The recording of the contraction force was performed in two moments: with the ankle in neutral position and in the position of maximum plantar flexion (Fig. 2 and 3). The illustration subtitles were elaborated to provide a clear and reproducible description of the methods and equipment used, ensuring that other researchers can accurately replicate the procedure.

After data collection, the analysis was performed, in which the variables between the groups were compared; an α = 0.05 and β = 0.2 were used. The statistical analysis methodology was performed using the *R programming language.* A definition of normality was made through graphical analysis and Shapiro-Wilk test. For the descriptive analysis, the quantitative variables with normal distribution were represented by their mean and standard deviations, those of non-normal distribution per quartile (Q_1/4_, median, Q_3/4_).

Comparisons between groups were made through the Kruskal-Wallis test for non-normal distribution variables and ANOVA test for normal distribution variables.

## RESULTS

**Figure 4:**
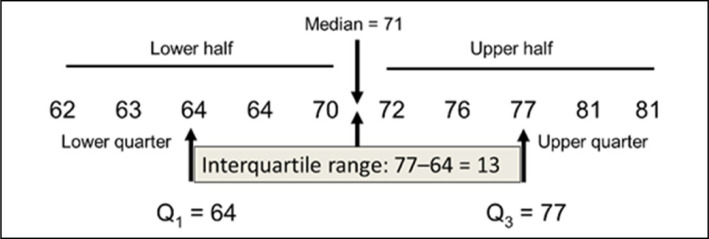
Median and Interquartile ranges.

After data collection, tabulation of the data was carried out for further analysis. Therefore, the definition of median and interquartile ranges can be observed in Figure 4.

The data obtained can be observed in Table 1, which contains the results obtained after clinical evaluation and physical examination of patients. The age variable is expressed in years; the rest correspond to the force in Newtons. The interpretation of the table takes into account the distribution for each group; normal variables present average and standard deviation (XX ± YY), non-normal numeric variables present median and lower and upper quartile (XX (Inf – Sup).

**Table 1:** Data obtained after clinical evaluation and physical examination of patients.

Variable/Group	Total (n = 63)	With Diabetic Neuropathy (n = 20)	Non-Neuropathic Diabetic Patients (n = 23)	Healthy Controls (n = 20)	P value
AGE	66.0(62.5-68.0)	66.0(64.8-69.0)	66.0(65.0-68.0)	63.0(61.0-67.2)	0.226
Hallux (Right) – Neutral Position	4.5±2.3	3.6 ± 1.7	4.8 ± 1.6	5.2 ± 3.1	0.054
Right Toes – Neutral Position	4.0±1.5	3.6 ± 1.2	4.6 ± 1.7	3.8 ± 1.5	0.066
All Toes (Right) – Neutral Position	5.5(3.5-6.8)	3.7(3.2-5.4)	6.1(3.8-6.9)	5.9(4.8-6.9)	0.024
Right Hallux – Plantar Flexion	2.9(2.0-3.8)	2.8(1.8-3.5)	3.0(2.2-3.9)	3.0(2.2-4.1)	0.468
Right Toes – Plantar Flexion	2.9(2.0-3.8)	2.4(1.8-3.1)	2.9(2.1-4.0)	3.3(2.2-4.2)	0.072
All Toes (Right) – Plantar Flexion	3.8±1.8	3.0 ± 0.9	4.0 ± 1.7	4.3 ± 2.2	0.045
Left Hallux – Neutral Position	3.2(2.5-4.8)	3.0(2.5-4.7)	3.2(2.5-4.8)	3.7(2.6-4.7)	0.790
Left Toes – Neutral Position	4.5±1.7	4.6 ± 1.8	4.6 ± 1.9	4.4 ± 1.6	0.901
All Toes (Left) – Neutral Position	5.2±2.1	4.9 ± 2.0	5.4 ± 2.3	5.2 ± 2.2	0.746
Left Hallux – Plantar Flexion	2.4(1.8-3.2)	2.4(2.0-2.8)	2.1(1.8-2.6)	2.8(2.0-4.1)	0.244
Left Toes – Plantar Flexion	2.9(2.1-4.2)	2.5(2.1-3.4)	2.9(2.0-4.4)	3.4(2.7-4.8)	0.054
All Toes (Left) – Plantar Flexion	3.0(2.3-4.4)	2.8(2.2-3.8)	3.0(2.3-4.2)	4.0(2.8-5.1)	0.082

The results of the statistical tests are given by the column ‘Value of p’. Values below 0.05 show statistically significant difference between groups, i.e., the groups present different results among themselves. Values greater than 0.05 tells us that the groups are statistically similar.

**Figure 5:**
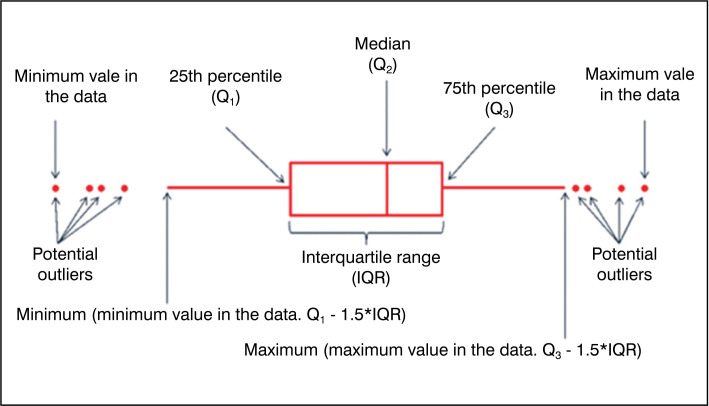
Anatomy of a Boxplot chart.

In addition, graphs of the type *Boxplot* were elaborated – which has its characteristics presented in Figure 5 for data analysis.

**Figure 6:**
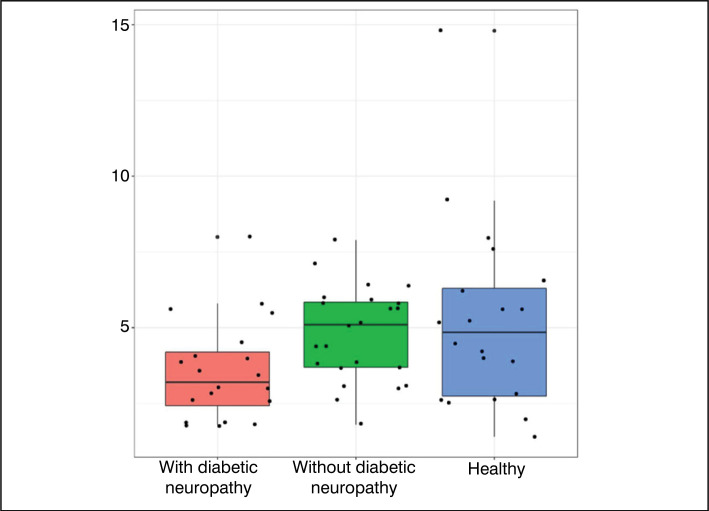
Results obtained for the Right Hallux (Neutral Position).

**Figure 7:**
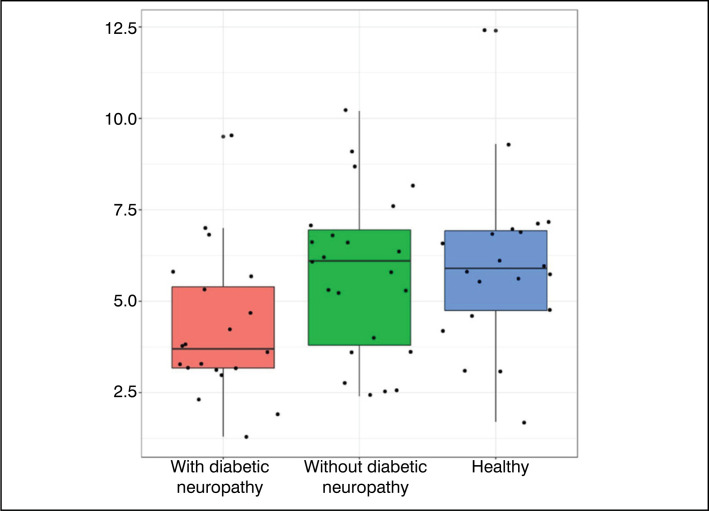
Results obtained for the variable All Toes (Right) – Neutral Position.

**Figure 8:**
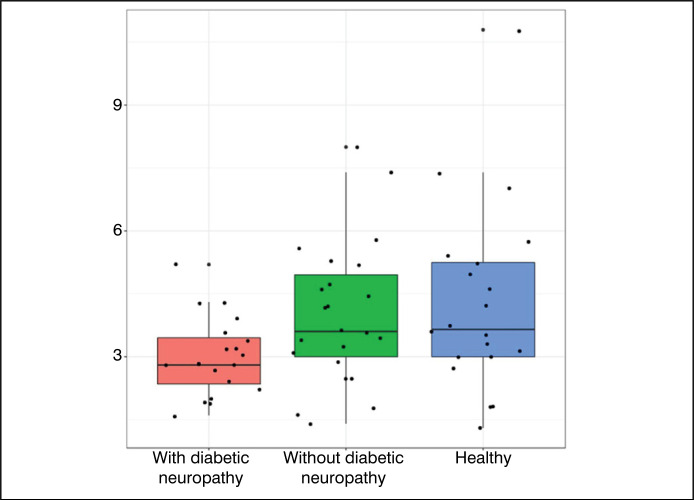
Results obtained for the variable All Toes (Right) – Plantar Flexion.

**Figure 9:**
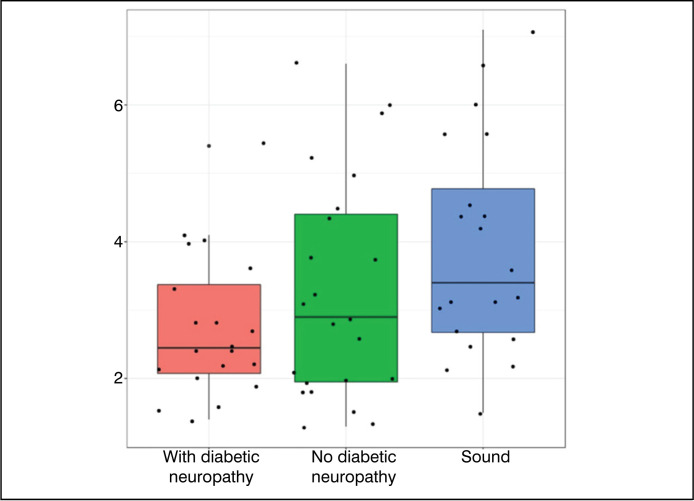
Results obtained for the variable Left Toes – Plantar Flexion.

Figures 6 to 9 were constructed in the *Boxplot* format, and used the variables of statistically significant value (p<0.05) – with the exception of the variable ‘Hallux R - neutral’ and ‘Toes L – plantar flexion’.

## DISCUSSION

Studies indicate that diabetes *mellitus* may result in loss of muscle strength, especially in the lower limbs, leading to a decline in physical function and reduction in the quality of life of patients.^14^ This loss of strength is not limited to the lower limbs, but may also affect the strength of the hand, suggesting a diffuse loss of strength among diabetic patients.^15^

### Intrinsic Foot Muscle Anatomy

The human foot is an adaptable structure, designed to adjust to surface and load variations, keeping efficient force transmission between the lower limb and the soil. The complex interaction of movements in the small joints of the foot allows the adaptation of the longitudinal arc during the walk, absorbing and reusing forces such as elastic energy.^16^ The plantar aponeurosis and the windlass mechanism are essential for foot stiffness during walking and for efficient propulsion.^16^ The intrinsic musculature of the foot, organized in four layers, plays a crucial role in the maintenance of the structural and functional integrity of the foot.^16,17^

### Strength Analysis of Intrinsic Foot Musculature

Deficits in the strength of the intrinsic muscle of the foot are associated with various pathologies and compromise the balance of the lower limbs. Weakness or altered activation of this muscle can contribute to conditions such as cavus foot, plantar fasciitis, claw toe deformities, hammertoe, retraction of the medial longitudinal arch, hallux valgus, and post-medial ankle pain.^18^ In diabetic patients, these deformities, combined with sensory neuropathy, increase the risk of plantar ulcers due to pressure in the affected areas.^19^

The evaluation of the intrinsic muscular strength of the foot is important, even in the presence of diabetic neuropathy, which initially manifests itself as a sensitive condition. Measurement of muscle strength can identify early changes in muscle function that are not detectable only by sensitive symptoms. The loss of muscle strength can precede and aggravate complications, offering an opportunity for preventive interventions before the deformities and ulcers develop. Therefore, intrinsic muscle strength can provide valuable insights about the progression of neuropathy and the risk of related complications.

### Evaluation Method and Results

To evaluate muscle strength in the lower limbs accurately and reliably, objective techniques such as dynamometry are preferable to manual muscle testing, which has limitations in sensitivity and reliability.^20^ Dynamometry provides quantitative and reproducible measurements, although different types of dynamometers may present variations in muscle strength results.^21^ It is important to note that the dynamometers do not activate the muscle; activation is performed by the patient during the test. Studies suggest that by keeping the ankle at maximum plantar flexion, the influence of the extrinsic flexors of the fingers is minimized, allowing for a more accurate evaluation of the intrinsic muscles.^22,23^

Data analysis (Table 1) showed that most variables did not present statistically significant differences between the groups. However, the variables Right Hallux – Neutral Position, All Toes (Right) – Neutral Position, All Toes (Right) – Plantar Flexion, and Left Toes – Plantar Flexion showed statistically significant differences. The comparative graphs (Figures 1–4) corroborate these findings, showing lower median values and narrower interquartile ranges in the groups with diabetic neuropathy.

The data from our study showed that patients with diabetes showed a 14% reduction in the strength of the flexors and 17% in the ankle extensors, plus a decrease in the strength and volume of the intrinsic muscle of the foot.^24^ Although these results are indicative, they are not conclusive as to the increased risk of reduction in the strength of the intrinsic muscle of the foot in patients with diabetic neuropathy. Limitations of the study, such as the size of the sample and the lack of data on the time of disease progression and markers of clinical severity, restrict the more robust interpretation of the results.

## CONCLUSION

This study sought to explore the strength of the intrinsic musculature of the foot in diabetic patients, using dynamometry as an evaluation tool. Although no conclusive evidence has been found to support the initial hypothesis, the data suggest that dynamometry can play a valuable role in the early detection of muscle weakness in patients with diabetic neuropathy. However, the limited size of the sample and the lack of detailed information on the time of disease progression and markers of clinical severity are limited to the generalization of the results.

It is concluded that this work establishes a basis for future research in the area, proposing a methodology that, with improvements, can contribute to the monitoring of muscle deterioration and the effectiveness of specific treatments in diabetic patients. It is considered essential to enlarge the study, with a larger sample and additional variables, such as disease progression time and progression markers, to validate and refine the preliminary conclusions, with potential to provide an early diagnostic marker of peripheral complications related to diabetes.

## Data Availability

The contents underlying the research are available in the manuscript.
